# Supplementary material for: Analysis of the Mouse Hepatic Peroxisome Proteome—Identification of Novel Protein Constituents Using a Semi-Quantitative SWATH-MS Approach
Source: Cells. 2024 Jan 17;13(2):176. doi: 10.3390/cells13020176 (PMC10814758; doi:10.3390/cells13020176)
Supplement: Supplementary file 1 [file cells-13-00176-s001.zip › Fig. S1.pdf]

A

B

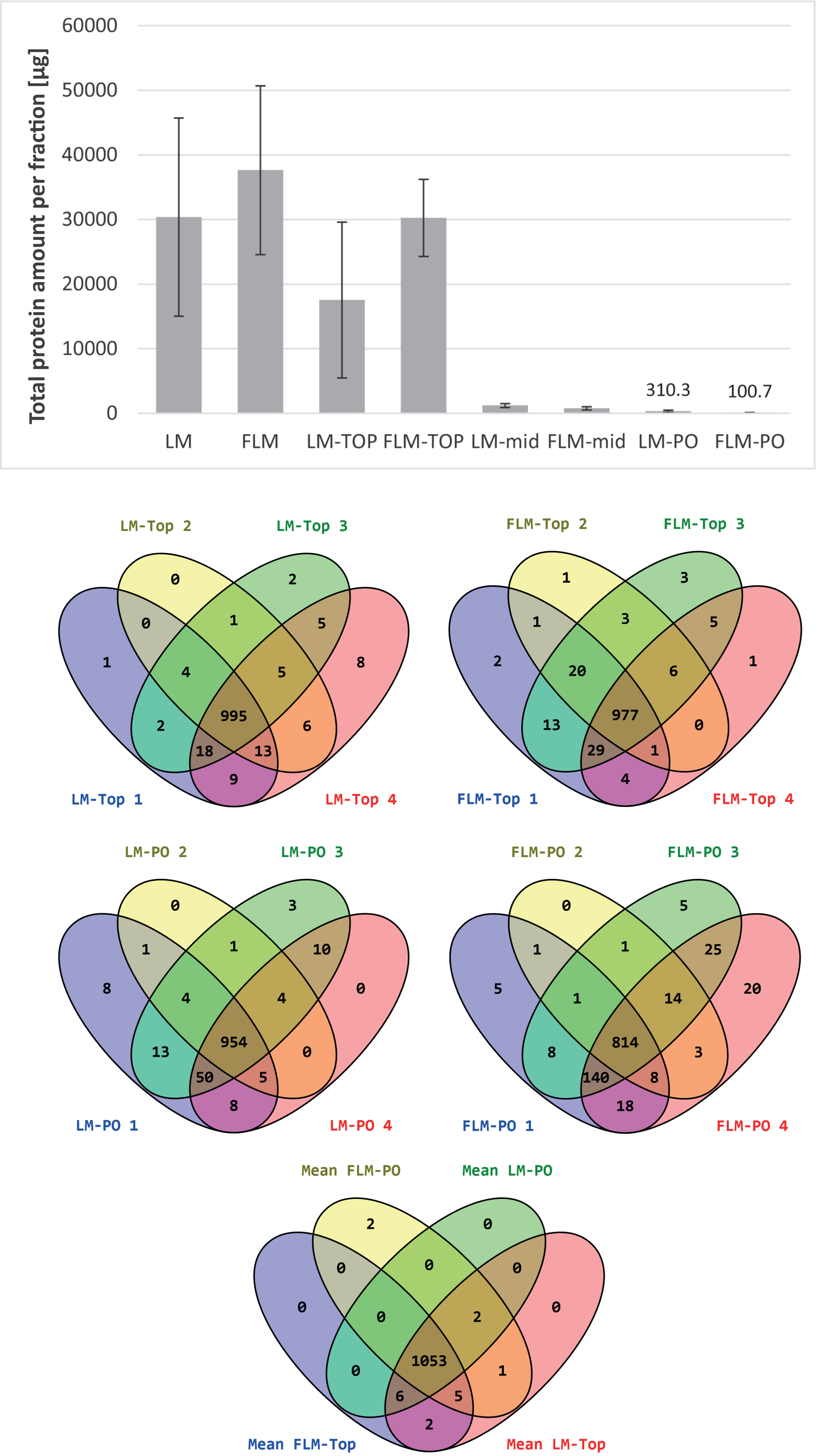

Fig. S1: (A) Average total protein amounts of the fractions subjected to MS. The graph compares prefractions subjected to density gradient centrifugastion (LM, FLM) with the fractions eluted from the iodixanol gradients (LM-TOP, FLM-TOP, LM-PO, FLM-PO), which were analyzed via SWATH-MS. LM-mid/FLM-mid corresponds to the material, which was eluted in between the analyzed fractions.

(B) Overlap of quantified proteins among the replicate SWATH-MS runs and means of the 4 experimental groups, repectively. For ≥ 90% of the proteins the qantification is based on at least 3 replicates (LM-TOP 96%, FLM-TOP 94%, LM-PO 94%, FLM-PO 90%).
