# Supplementary material for: Analysis of the Mouse Hepatic Peroxisome Proteome—Identification of Novel Protein Constituents Using a Semi-Quantitative SWATH-MS Approach
Source: Cells. 2024 Jan 17;13(2):176. doi: 10.3390/cells13020176 (PMC10814758; doi:10.3390/cells13020176)
Supplement: Supplementary file 1 [file cells-13-00176-s001.zip › Fig. S2.pdf]

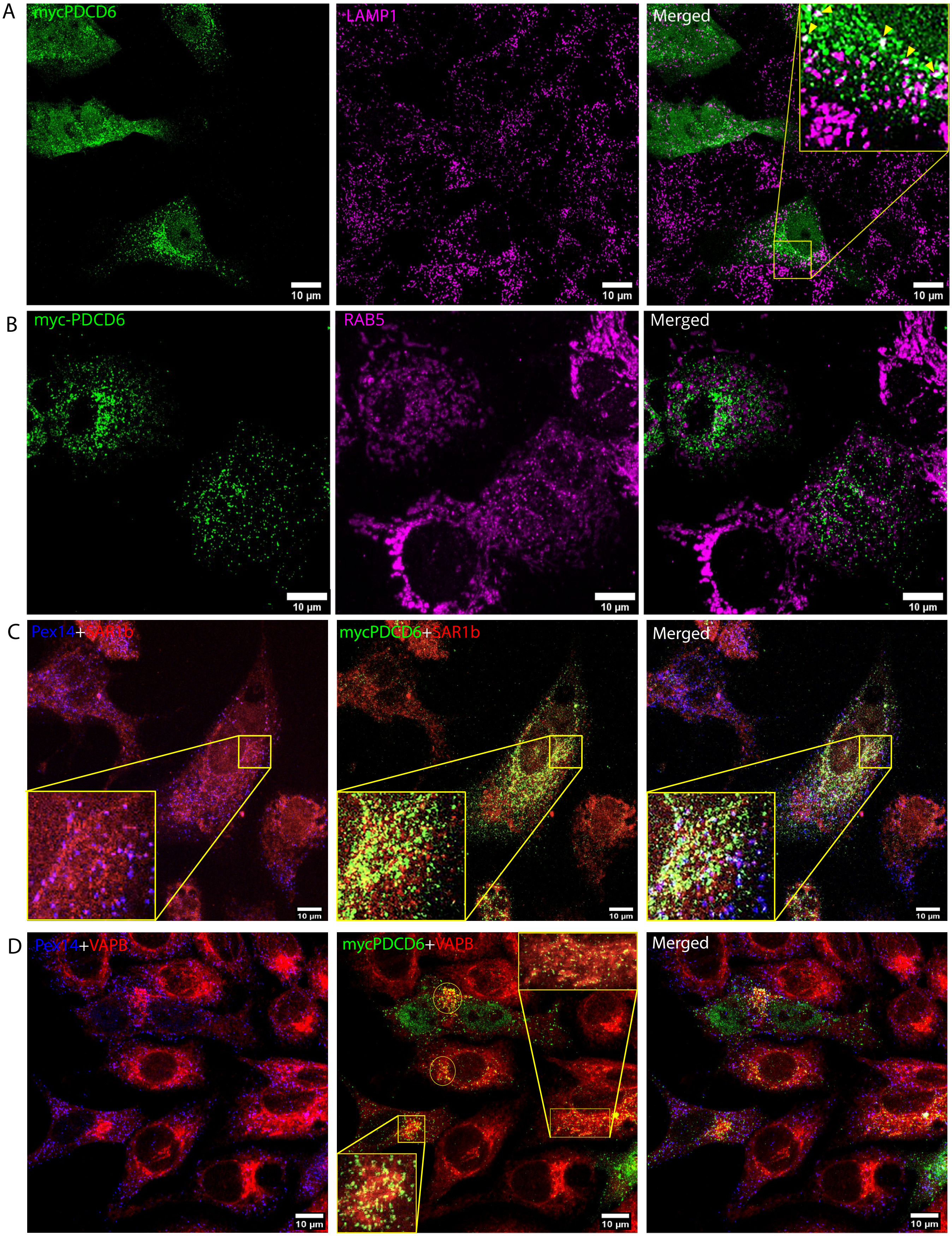

Suppl. Fig. S2: Immunofluorescence colocalization analysis of overexpressed myc-PDCD6 (green) with (A) the late endosome marker LAMP1 (magenta), (B) the early endosome marker RAB5 (magenta), the ERES markers (C) SAR1B (red) and (D) VAPB (red). In (C) and (D) peroxisomes are marked by PEX14 (blue).
