# Supplementary material for: Analysis of the Mouse Hepatic Peroxisome Proteome—Identification of Novel Protein Constituents Using a Semi-Quantitative SWATH-MS Approach
Source: Cells. 2024 Jan 17;13(2):176. doi: 10.3390/cells13020176 (PMC10814758; doi:10.3390/cells13020176)
Supplement: Supplementary file 1 [file cells-13-00176-s001.zip › Suppl. Tables S1-3.pdf]

Table S1: List of primary antibodies

Target	Source	Immunogen	Dilution	Host Species	Application
**ABCD3**	Sigma-Aldrich, SAB4200181 Schnelldorf, Germany	ATP-binding cassette (ABC) transporter	1:1200	Mouse mAb	WB
**ACBD5**	Abcam (ab100910) Cambridge, UK	Acyl-CoA-binding domain-containing protein 5	1:1000	RabbitpAb	WB
**ACAD11**	Gift from G. Vockley, Pittsburg University, USA	Acyl-CoA dehydrogenase 11	1:2000	Rabbit pAb	WB
**ACOX1**	Gift from T. Hashimoto, Shinshu University School of Medicine, Nagano, JP	Peroxisomal acyl-coenzyme A oxidase 1	1:10.000	Rabbit	WB
**ATPA**	BD Transduction Laboratories 612517 San Diego, USA	ATP synthase subunit alpha, mitochondrial	1:10.000	Mouse mAb	WB
**ATPB**	Proteintech (66600) Rosemont IL, USA	β-subunit of ATP synthase	1:5000	Mouse mAb	WB
**BiP/GRP78**	BD Transduction Laboratories 610978 San Diego USA	Endoplasmic reticulum chaperone BiP	1:1000	Mouse mAb	WB
**Catalase**	Gift from A. Völkl, University of Heidelberg, Germany	Catalase	10 ug/ml	Rabbit	WB
**c-myc tag (9B11)**	Cell Signaling #2276 Danvers, Massachusetts USA	myc epitope tag	1:500	Mouse mAb	IF
**ERP29 (LY1C6)**	Abcam ab11420-50 Cambridge, UK	Endoplasmic reticulum resident protein 29	1:5000	Rabbit pAb	WB
**FATP4**	Abcam ab200353 Cambridge, UK	Fatty acid protein 4	1:1000	Rabbit mAb	WB
**LAMP1**	Santa Cruz Biotechnology sc-17768 Santa Cruz,USA	Lysosome-associated membrane proteins	1:500	Mouse mAb	IF
**OCIAD1**	Novus biotechne NBP1-76242 Abingdon UK	OCIA-domain containing protein 1	1:100	Rabbit pAb	IF
**PEX3**	Gift from G. Dodt, University of Tübingen, Germany	Peroxisomal Biogenesis Factor 3	1:1000	Rabbit	WB

**PEX14**	Proteogenix,selfmade (7790-03107-A02) Oberhausbergen, France	Peroxisomal Biogenesis Factor 14	1:2000	Guinea pig	IF
**PMP22**	Gift from A. Völkl, University of Heidelberg, Germany	Peroxisomal membrane protein 22	1:500	Rabbit	WB
**RAB5A**	Proteintech 11974-1-AP Rosemont IL, USA	The small GTPase Rab	1:1000	Rabbit pAb	IF
**SAR1B**	Proteintech/ptglab 22292-1-AP Rosemont IL, USA	Secretion Associated Ras Related GTPase 1B	1:200	Rabbit pAb	IF
**SCP-X**	MyBio Source MBS 1495100 San Diego, USA	Serol Carrier Protein	1:500	Rabbit pAb	WB
**TOMM20**	BD Transduction Laboratories 612278 San Diego, USA	Translocase of the Outer Membrane of the mitochondria	1:1000	Mouse mAb	WB, IF

Table S2: List of secondary antibodies

Conjugated Molecule	Source	Dilution	Reactiviy	Use
**Alexa Fluor® 488**	Invitrogen A32723	1:1000	Goat anti mouse IgG	IF
**Alexa Fluor® 488**	Invitrogen A32723	1:1000	Goat anti mouse IgG	IF
**Alexa Fluor® 568**	Invitrogen A11041	1:1000	Goat anti chicken IgG	IF
**Alexa Fluor® 568**	Invitrogen A11011	1:1000	Goat anti rabbit IgG	IF
**Alexa Fluor® 568**	Invitrogen A11036	1:1000	Goat anti rat IgG	IF
**Alexa Fluor® 647**	Invitrogen A21450	1:500	Goat anti guinea pig IgG	IF
**Alexa Fluor® 647**	Invitrogen A32733	1:500	Goat anti rabbit IgG	IF
**HRP IgG**	DAKO (P0447),	1:5000	Goat anti mouse	WB
**HRP IgG**	DAKO (P0448),	1:5000	Goat anti rabbit	WB

Table S3: Primer list

Gene	Forward	Reverse	Product Size	Tm
m**PDCD6**	CTTGGATCCCATGGCTGCCTACTCCTACCG	GGGAAGCTTTTATACAATGCTGAAGACC	573 base pair	66 °C
m**OCIAD1**	CTTGAATTCGATGAATGGGAGGGCTG	CCTCTCGAGTTACTCATCCCAAGTATC	1529 base pair	64 °C
m**PAFAH2**	CTTGAATTCGATGGGGGCCGGCCAGT	GGGAAGCTTCTATAGACTAGACAGATAGTGCGG	1173 base pair	66 °C
m**HTATIP2**	CTTGAATTCGATGGCGGACAAGGAAGC	CCTCTCGAGTTATCACAGTTTGGGC	729 base pair	66 °C
