# Supplementary figures and images for: Analysis of the Mouse Hepatic Peroxisome Proteome—Identification of Novel Protein Constituents Using a Semi-Quantitative SWATH-MS Approach
Source: Cells. 2024 Jan 17;13(2):176. doi: 10.3390/cells13020176 (PMC10814758; doi:10.3390/cells13020176)

A

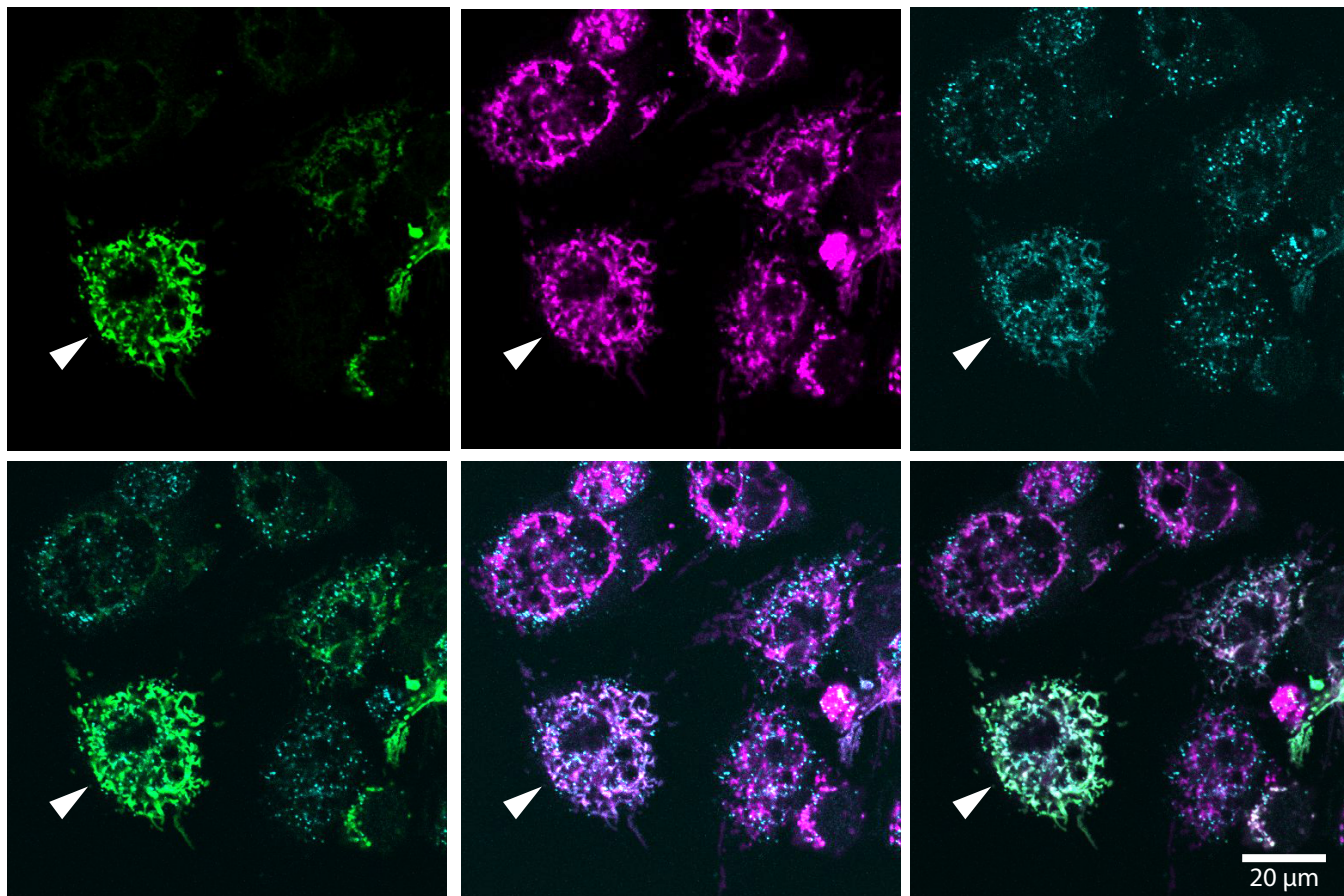

B

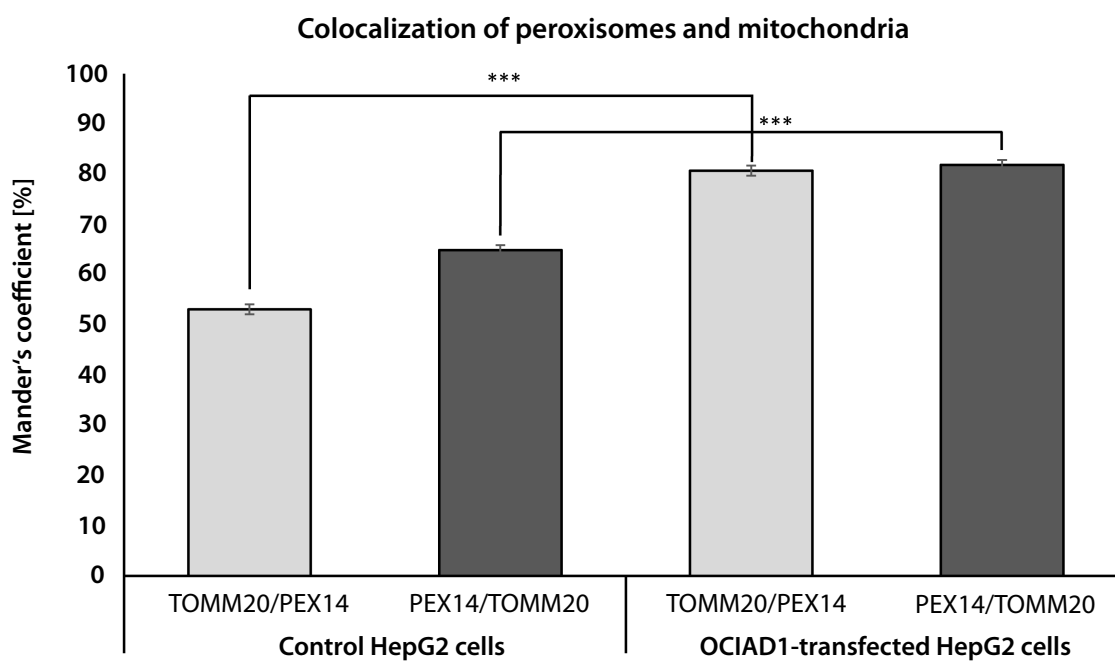

Supplement: Supplementary file 1 [file cells-13-00176-s001.zip › Fig. S3.pdf]

Original immunoblots from Fig. 2A:

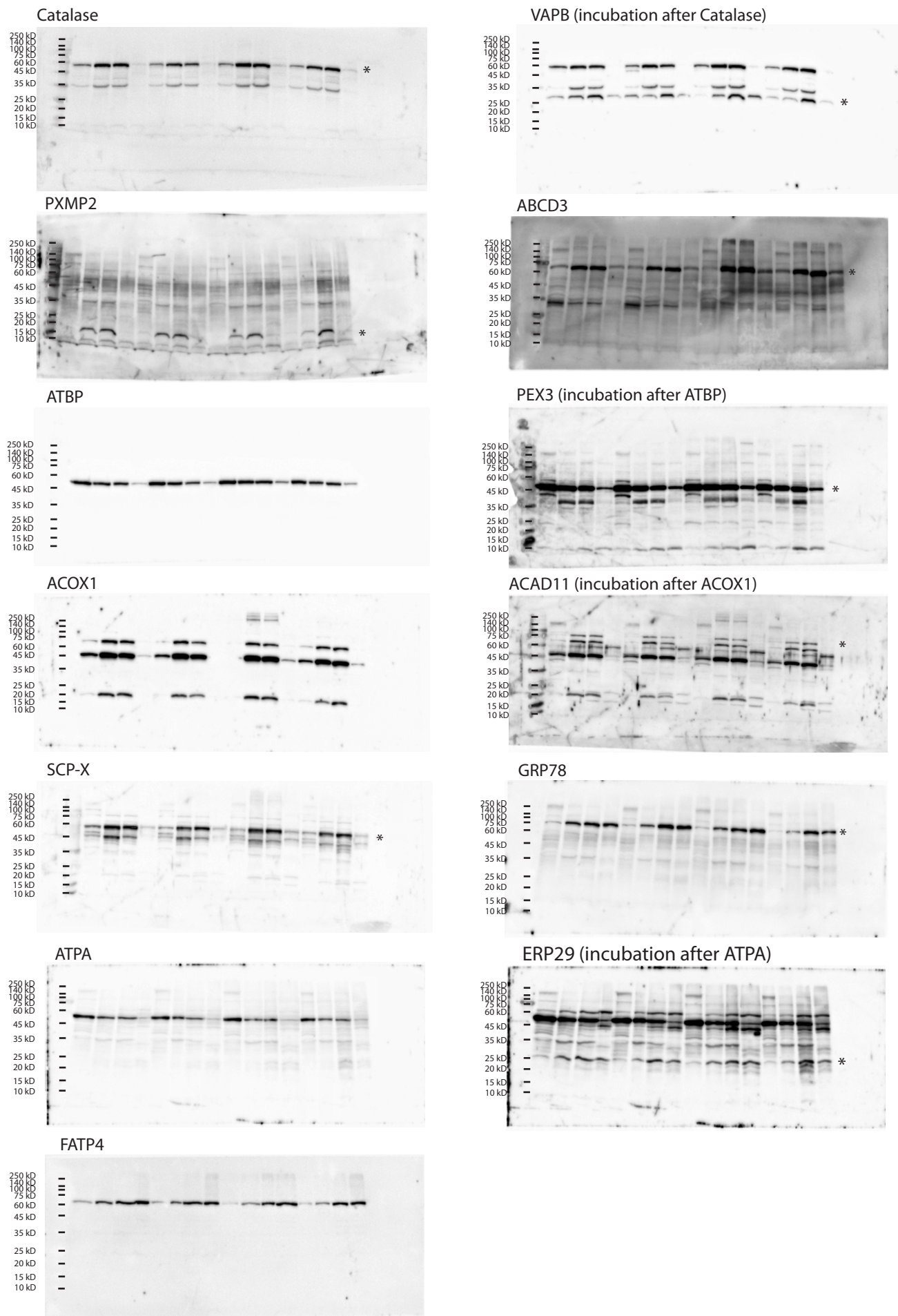

Supplement: Supplementary file 1 [file cells-13-00176-s001.zip › WBs Fig. 2A_rev.pdf]

Original immunoblots from Fig. 2B

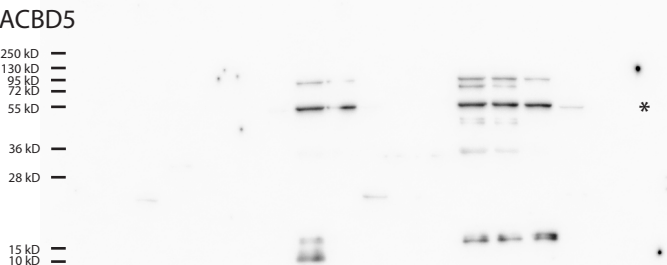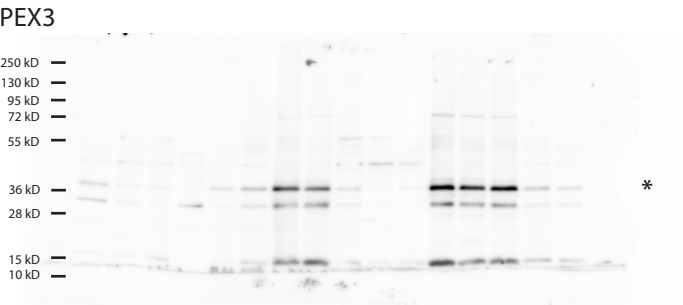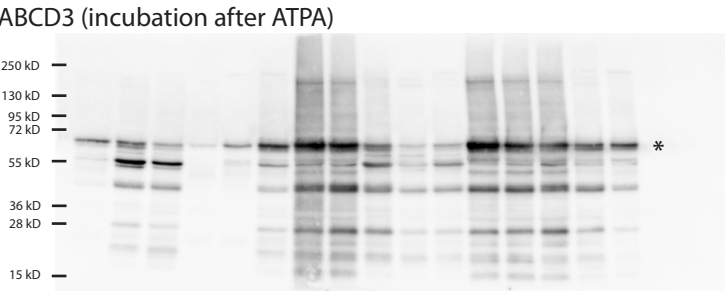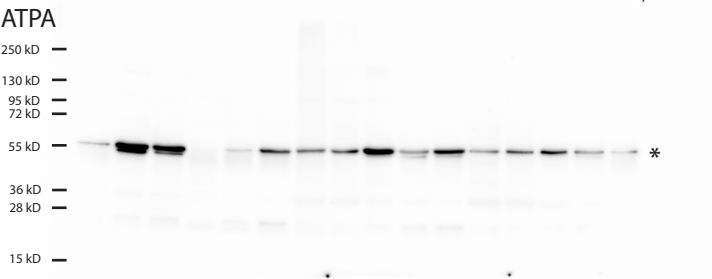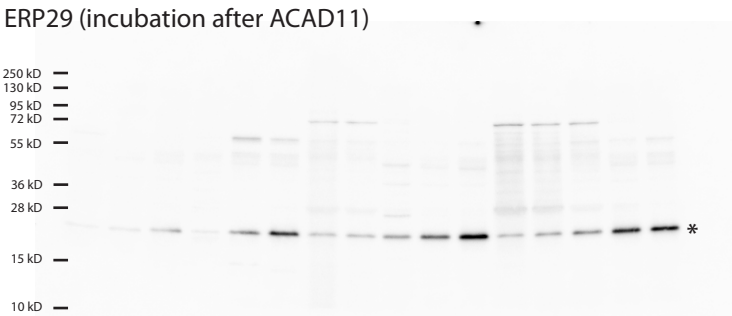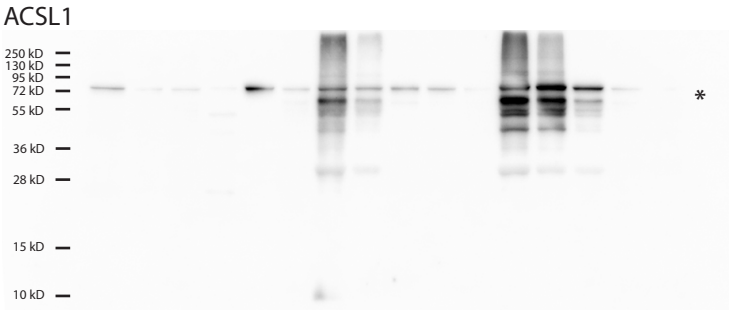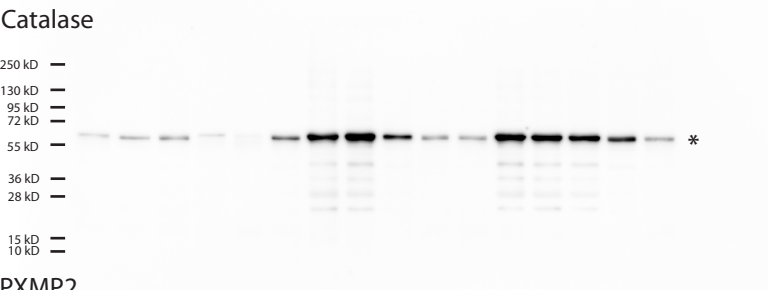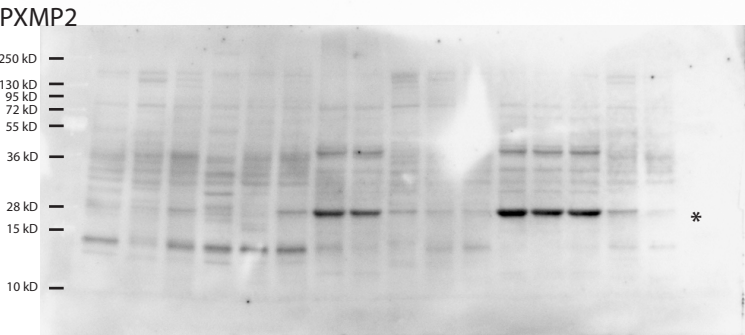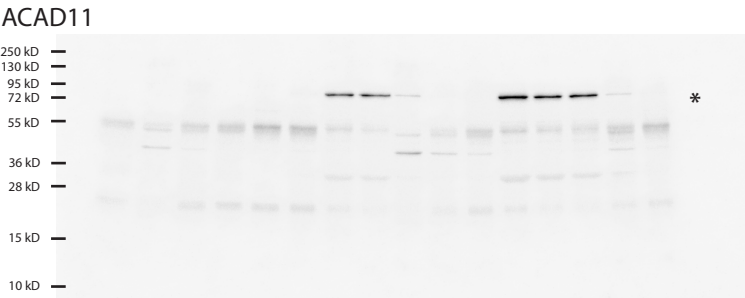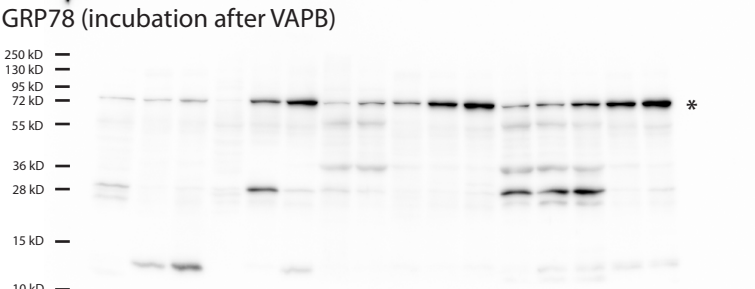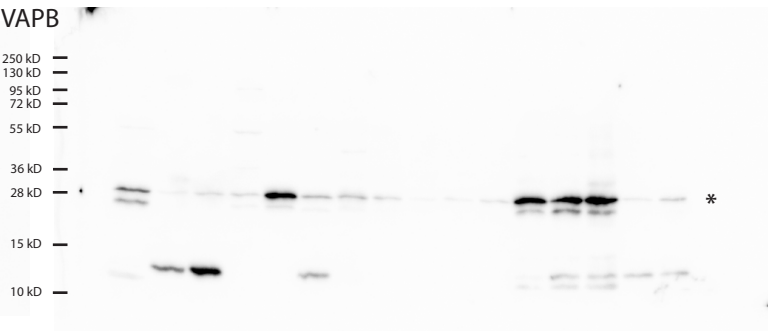

Supplement: Supplementary file 1 [file cells-13-00176-s001.zip › WBs Fig. 2B_rev.pdf]
